# Case report and literature review: Laparoscopic extended right hemicolectomy for a 55-year-old patient with idiopathic mesenteric phlebosclerosis

**DOI:** 10.3389/fmed.2024.1382475

**Published:** 2024-07-16

**Authors:** Siyu Liu, Yujun Tong, Ruizi Shi, Xintao Zeng, Hua Luo, Pei Yang, Xianfu Cai, Decai Wang, Huiwen Luo, Jianjun Wang

**Affiliations:** ^1^Department of Breast Surgery, Mianyang Central Hospital, School of Medicine, University of Electronic Science and Technology of China, Mianyang, China; ^2^Department of Hepatobiliary Surgery, Mianyang Central Hospital, School of Medicine, University of Electronic Science and Technology of China, Mianyang, China; ^3^Department of Urology, Mianyang Central Hospital, School of Medicine, University of Electronic Science and Technology of China, Mianyang, China; ^4^NHC Key Laboratory of Nuclear Technology Medical Transformation, Mianyang Central Hospital, School of Medicine, University of Electronic Science and Technology of China, Mianyang, China

**Keywords:** idiopathic mesenteric phlebosclerosis, laparoscopic surgery, case report, literature review, digestive disease

## Abstract

Idiopathic mesenteric phlebosclerosis (IMP) is an extremely rare disease with an unclear pathogenesis and risk factors. The clinical manifestations of IMP are mostly non-specific, mainly consisting of digestive symptoms such as abdominal pain, bloating and diarrhea. The diagnosis of IMP mainly relies on abdominal computed tomography (CT) and colonoscopy. Pathological changes associated with IMP often involve fibrous degeneration of the venous wall, which results in the thickening of the colonic wall and longitudinal calcification of the mesenteric arteries. Currently, there is no standard treatment protocol for IMP, and nonsurgical treatment is the mainstay of most medical centers. In this study, we reported a case of a 55-year-old female patient with IMP whose main clinical presentation was recurrent abdominal pain. The patient’s initial diagnosis was considered an incomplete intestinal obstruction and received non-surgical treatments; however, the efficacy of the treatment was unsatisfactory. After completing abdominal CT and colonoscopy, we excluded common diseases of the digestive system (e.g., tumors, Crohn’s disease), and finally considered that this patient had a high likelihood of IMP. This patient eventually underwent laparoscopic enlarged right hemicolectomy due to recurrent symptoms and poor outcomes of non-surgical treatment. Postoperative pathological results confirmed the diagnosis of IMP. During the follow-up period, the patient recovered well without recurrence of IMP. Furthermore, we have reviewed the literature related to IMP and summarized the etiology, risk factors, diagnostic methods, treatment options and prognosis of IMP.

## 1 Introduction

Idiopathic mesenteric phlebosclerosis (IMP) is an extremely rare clinical disease, the pathogenesis of which has not yet been clarified; its characteristic imaging manifestations and pathological findings are the main basis for clinical diagnosis. This disease was first reported in 1991, and in 2000, Yao et al. (1) named it “phlebosclerotic colitis” (PC) to distinguish it from common ischemic colitis. As the disease became better understood, Iwashita et al. (2) formally named it IMP in 2003, based on its pathophysiological characteristics as a non-specific inflammatory disease. IMP is often misdiagnosed owing to its atypical symptoms, such as abdominal discomfort, abdominal distension, changes in bowel movements, and intestinal obstruction. Most patients with IMP recover with non-surgical therapy such as nutritional assistance, inhibition of stomach acid production, and rehydration; a relatively few individuals with severe or persistent symptoms need surgical consultation. Here, we report the case of a female patient who presented to our hospital with recurrent abdominal pain. Initially, the patient was diagnosed with incomplete intestinal obstruction; however, after prolonged conservative treatment, the patient’s symptoms were not significantly relieved, and combined with the patient’s abdominal computed tomography (CT) and colonoscopy results, this rare diagnosis was considered. We performed laparoscopic extended right hemicolectomy in this patient, and the surgical procedure proceeded smoothly. The patient recovered well after the resection with resolution of her abdominal pain and other symptoms.

## 2 Case description

A 55-year-old patient presented to Mianyang Central Hospital with recurrent abdominal pain for 1 year, which became worse over 1 day. One year before admission, the patient had recurrent periumbilical abdominal pain with no obvious aggravating factors, accompanied by obstipation, and was initially diagnosed with an incomplete intestinal obstruction. After symptomatic treatment with antacids and rehydration, the patient was discharged from the hospital after her condition improved. During this year, the patient was admitted to hospital several times for the same symptoms, all of which resolved with nonsurgical treatment.

The day before this admission, the patient had recurrent abdominal pain with nausea and vomiting and denied taking any herbs or other medications. The patient did not have any other symptoms except for the digestive clinical manifestations. Physical examination revealed only a slight pain with palpation on the right side of the abdomen. Considering the recurrent periumbilical abdominal pain with defecation difficulties, we initially considered that the patient had a high probability of being diagnosed with incomplete intestinal obstruction. In order to relieve the patient’s abdominal pain and other symptoms as well as to maintain the body’s water-electrolyte balance, we performed symptomatic supportive treatments such as fasting, inhibiting gastric acid secretion, and rehydrating fluids, etc. During the treatment period, the patient’s symptoms were recurring, and the conservative treatment was not effective, therefore, we also considered the possibility of other gastrointestinal diseases, such as tumors, ulcerative colitis, and Crohn’s disease. Certainly, we also considered the possibility of rare diseases such as IMP. For definitive diagnosis, we further performed laboratory tests, abdominal CT, and colonoscopy.

**Figure 1:**
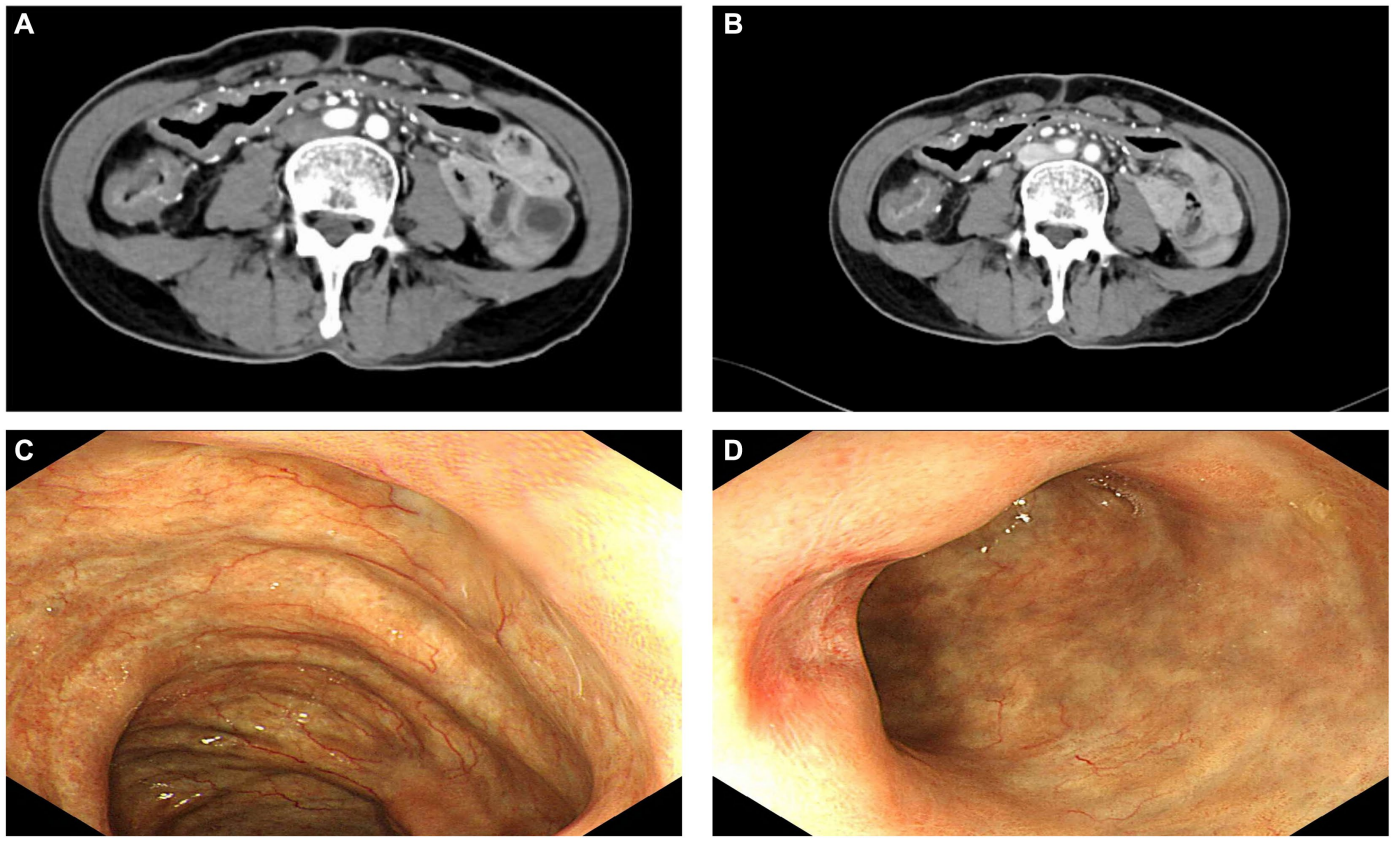
Preoperative abdominal computed tomography **(A,B)** and colonoscopy results **(C,D)**.

Laboratory test results were as follows: white blood cells 17.79 × 10^9/L, lymphocytes 0.69 × 10^9/L, neutrophils 16.6 × 10^9/L, hemoglobin 104 g/L, platelets 190 × 10^9/L, total protein 65.09 g/L, albumin 37.43 g/L, total bilirubin 7.8 μmol/L, blood urea nitrogen (BUN) 6.63 mmol/L, creatinine 44.7 μmol/L, aspartate amino transferase (AST) 31 U/L, alanine transaminase (ALT) 23 U/L, carcinoembryonic antigen (CEA) 0.96 ng/mL, alpha fetoprotein (AFP) 5.68 U/L, cancer antigen (CA) 125 12.81 U/mL, CA 19–9 8.9 U/mL, and human epididymis protein 4 (HE4) 39.49 pom/L. Abdominal contrast-enhanced CT revealed thickening of the walls of the right colon and transverse colon, with extensive calcification of the draining vessels and enlarged mesenteric lymph nodes (Figure 1). Colonoscopy showed that the right and transverse colonic mucosa was rough with dark copper-colored pigmented classes, vascular texture was lost, scattered capillary dilatation was observed, and the intestinal wall was stiff and rigid. In the ileocecal region, the mucosa was rough, dark copper-pigmented classes, the vascular texture was lost, scattered capillary dilatation was observed, and the intestinal wall was stiff and rigid (Figure 1).

Based on the patient’s medical history, laboratory findings, and abdominal CT and colonoscopy results, we believe that the patient had a high probability of having IMP. After a long period of conservative treatment, the patient’s abdominal pain and intestinal obstruction did not improve, and her general nutritional status deteriorated. Moreover, the patient experienced moderate anaemia (haemoglobin: 89 g/L) and hypoproteinaemia (albumin: 30.88 g/L) during the treatment period. Based on the results of abdominal CT and colonoscopy, and considering the potential recurrence of IMP, we performed laparoscopic enlarged right hemicolectomy for this patient after excluding contraindications to surgery.

Details of the surgical procedure are as follows. We selected the classic medial approach to free the right hemicolon, i.e., pulling the transverse colon upward, incising from below the ileocolic vessels, and bluntly separating to find the Toldt fascia layer, where the ileocolic artery was clamped and severed. Subsequently, we continued to free the Toldt gap cephalad to reveal the pancreatic neck. The neck of the pancreas was used as an anatomical landmark to locate the right colonic vessels and Henle’s trunk, which were clamped at the root of the vessels in the colon and then severed. The mesentery of the transverse colon was incised along the surface of the pancreas to both sides, exposing the right gastric omental vein, which was dissected from its root and dissected at the origin of the gastroduodenal artery below the pylorus. The Toldt’s fascia continued to expand outward and upward to free the mesentery of the ascending colon and the right mesentery of the transverse colon, and after turning upward to dissect the greater omentum, the right hemicolon was completely freed in the ileocecal region, and in the peritoneal dissection on the lateral side of the ascending colon. Thereafter, the transverse colonic mesentery on the surface of the pancreas was isolated, and a vascular clip at the root of the inferior mesenteric vein was clamped and severed to completely free the splenic region of the colon. The ileum and the corresponding ileocecal mesentery were cut at a distance of about 10 cm from the ileocaecum, and the transverse colon and its mesentery were cut at the splenic flexure of the colon. A hole is made in the mesenteric margin at the severed end of the descending colon and 5 cm from the severed end of the ileum, and the head of the intraluminal linear cutting closure is inserted into the opening of the ileum/ descending colon; the closure is slowly advanced until the opening of the ileum/descending colon reaches the jaws of the closure and is aligned; the anastomosis is slowly activated and removed. During this period, the assistant applied suction promptly using a suction device to avoid leakage of bowel contents into the abdominal cavity. Haemostasis was achieved by electrocoagulation. After the anastomosis was free of bleeding the common opening and the mesenteric fissure were closed with continuous sutures using absorbable barbed sutures, the specimen was removed and the incision was closed with layer-by-layer sutures. Moreover, we removed the enlarged lymph nodes in the mesentery during the procedure. Notably, we had more difficulty than before in exposing the surgical area due to the hard texture of part of the bowel wall.

**Figure 2:**
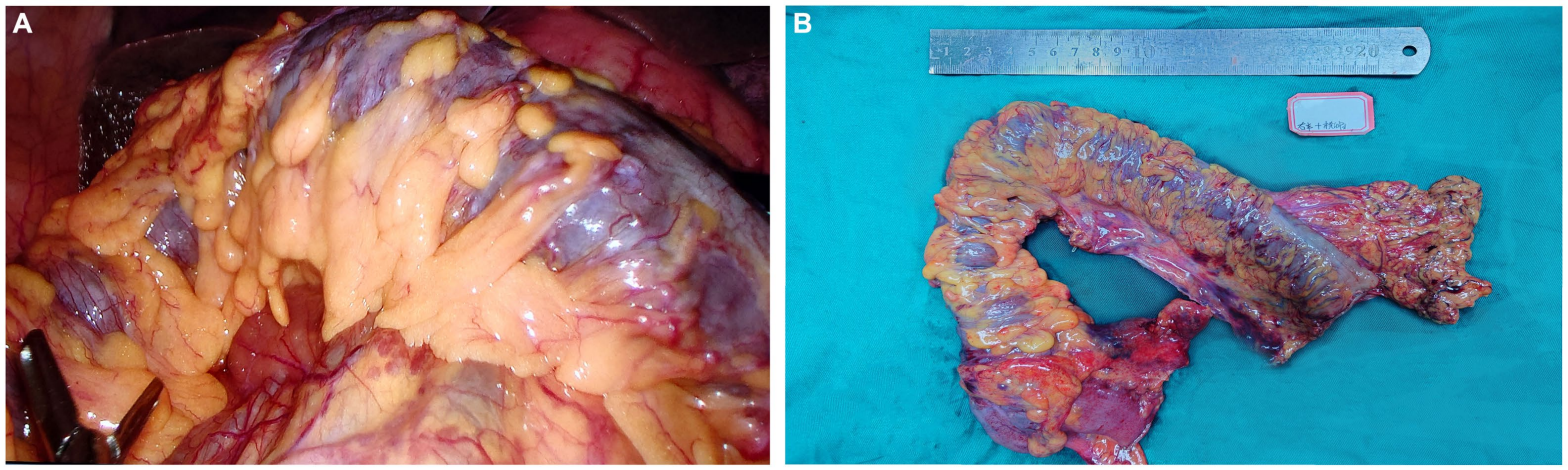
Intraoperative image **(A)** and the surgical specimen **(B)**.

Intraoperative and postoperative blood gas analyses revealed normal lactate levels and the patient had no significant acid–base imbalance. Dissection of the surgical specimen showed thickening of the intestinal wall up to 6 mm, disappearance of mucosal folds, and multiple enlarged lymph nodes in the peritoneal and mesenteric regions of the intestine, measuring approximately 0.5–1.0 cm in size and with a brittle texture. The venous vessels in the ascending and transverse colons were linear and hard (Figure 2).

**Figure 3:**
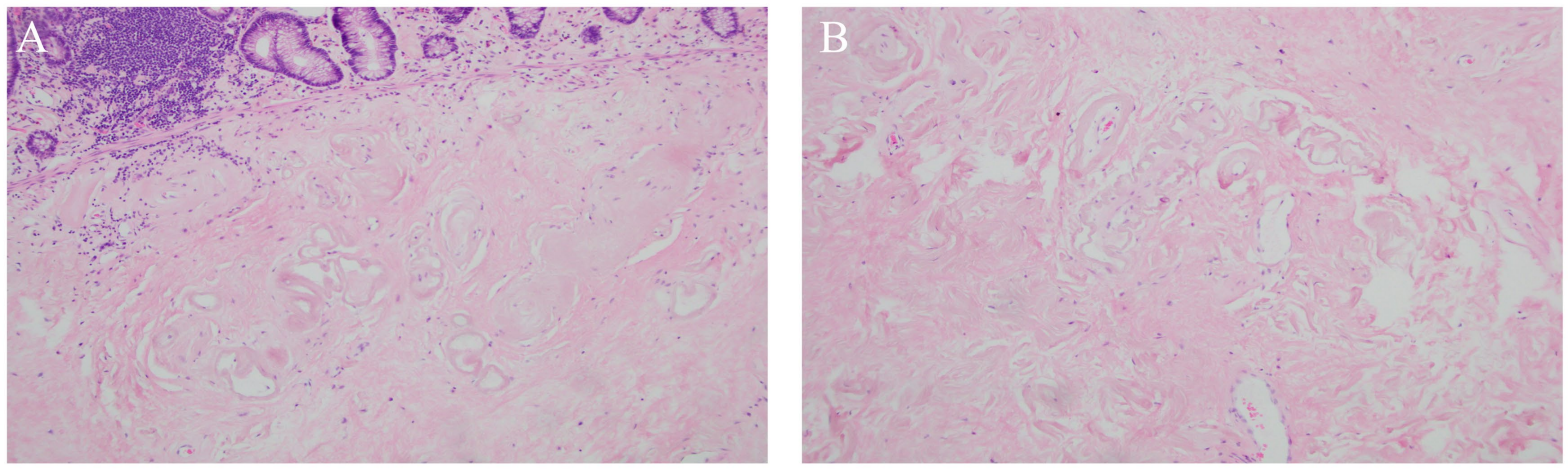
Postoperative histopathological results. **(A)** HE stain of colonic mucosa (magnification × 100); **(B)** HE stain of mesenteric vein (magnification × 100).

After standard post-operative care including antibiotics, nutritional support and rehydration, the patient recovered well and was discharged from the hospital. The postoperative pathological findings suggested chronic inflammation of the intestinal mucosa with focal degeneration, thickening of the submucosal layer, extensive hyaline lesions of the submucosal and intramuscular vessels, calcification in focal areas, and enlargement of the lumen of some vessels with wall thickening, hyaline lesions, and calcification (Figure 3). And the enlarged lymph nodes around the mesentery were confirmed to be reactive hyperplastic changes.

The patient was closely followed up after discharge. Symptoms such as abdominal pain subsided, the patient’s diet, sleep, and bowel movements were all normal, and laboratory results such as blood analyses, liver function, renal function, and electrolytes were all in the normal range. Regular postoperative abdominal CT examination also did not reveal any new sclerotic vessels.

## 3 Discussion

IMP is very rare in clinical practice, and the pathologic mechanism is currently unknown. It is now believed that IMP occurs primarily due to fibrotic degeneration of the venous wall leading to linear calcification along the mesenteric vessels and thickening of the colonic wall (3). We reviewed and summarized the risk factors for IMP. In previously reported cases of IMP, some patients had a long history of taking herbal medicines, especially those containing gardenia glycosides, so toxins and chemicals may be one of the risk factors for the development of IMP (4–6). Also, most patients with IMP are Asians, so geographic distribution, genetic predisposition, and idiosyncratic lifestyle may be involved in the development of the disease. Some scholars have also suggested that diseases such as right heart failure and portal hypertension can lead to prolonged elevation of venous pressure and adaptive changes in the venous walls followed by venous sclerosis (2, 7). Moreover, diabetes mellitus, dyslipidemia, cirrhosis and autoimmune diseases have also been suggested as potential causes of IMP (3, 8).

Abdominal CT is a reliable tool for diagnosing IMP, which manifests as calcification of the mesenteric vein and its branches. It has been suggested that patients with more extensive venous calcification are more likely to require surgical intervention and intestinal resection (8). Assessing the severity of IMP based on the total mesenteric vein calcification score, number of colon segments involved, and presence of dilated intestinal collaterals on CT may help determine the effectiveness of conservative management and the need for resection. The diagnosis of IMP is mainly based on abdominal CT, and combined with colonoscopy can improve the diagnostic yield. On CT, IMP mainly showed linear calcification of the mesenteric venous vessels and thickening of the colonic wall in the involved bowel segments, whereas colonoscopy mainly showed a dark purple or dark brown color, edema, and loss of semilunar folds on the surface of the colon (9). Microscopically, the colonic mucosa showed fibrosis, hyalinoid degeneration, and extensive thickening, whereas the venous wall showed fibrotic thickening, calcification, submucosal fibrosis, mucosal collagen deposition, and foamy macrophages in the vascular wall (9), consistent with the pathological findings reported in the present case.

IMP develops mainly in Asian populations, with a predominance of right colonic lesions, and its main clinical manifestations are characterized by nonspecific symptoms, such as abdominal pain, abdominal distension, nausea, vomiting, and constipation, which may be related to the severity of mesenteric venous sclerosis and calcification. IMP is mainly due to fibrous degeneration of the mesenteric vein wall leading to linear calcification along the mesenteric vessels and thickening of the colonic wall, so the main clinical manifestations are multiple gastrointestinal symptoms, which are mainly characterized by nonspecific symptoms such as abdominal pain, abdominal distension, nausea, vomiting, and constipation. We have carefully reviewed previous related cases and found no reports of extracolonic manifestations of IMP. A retrospective study in Japan showed that abdominal pain, distension, and diarrhea were the three most common symptoms of IMP (4). Therefore, it is difficult to make a clear diagnosis based on the clinical manifestations. In the present case, the patient was misdiagnosed with intestinal obstruction several times, which delayed the diagnosis.

To further explore and study IMP, we searched the database with the keywords “idiopathic mesenteric phlebosclerosis” (IMP) as well as “phlebosclerotic colitis” (PC) to retrieve a total of 78 case reports from 1997 to present. These 78 cases are further (Table 1). The average age of these patients was 53 years old (22–84 years old), with a male-to-female incidence rate of approximately 1:0.9, and only two cases were from non-Asian regions, which is consistent with previous reports (10, 11). Most patients (74.4%) had a history of long-term use of herbs or other medications. Some studies have shown that IMP is associated with chronic intake of biochemicals and toxins, and that long-term use of gardenia glycoside-containing herbs has been associated with the disease (4–6). Since the clinical manifestations of IMP are not specific, most patients are asymptomatic in the early stages. Among the 78 patients, 59 had abdominal pain (75.6%) as the first symptom, and most were misdiagnosed with intestinal obstruction. This case also involved multiple admissions for abdominal pain and was misdiagnosed as intestinal obstruction, which is consistent with previous reports. Currently, there is no guideline opinion and consensus on the treatment of IMP, and treatment is individualized and determined according to the severity of the patient’s condition. Patients with mild disease can be treated conservatively with follow-up. Among the 78 reported cases, 40 were treated conservatively, and most were able to improve their symptoms and histology after stopping the use of herbs and symptomatic management. Patients with severe disease (such as perforation, bleeding, and persistent intestinal obstruction) or unrelieved symptoms usually require surgery, usually a subtotal or total colectomy. Fourteen of these 78 reported cases were total colectomies, ten were right hemicolectomies, eleven were other hemicolectomies and ileostomies, all of which were seen to have dark purple intestinal mucosal with edema, disappearance of the mucosal folds, and sclerosis of the mesenteric vascularization during the operation. According to these reports, postoperative reviews and follow-ups suggest that IMP surgical cases generally have a good prognosis with no recurrence. Here, we report a case of IMP misdiagnosed as intestinal obstruction and describe the necessity of surgical resection of the diseased bowel in cases where conservative treatment is ineffective. Furthermore, the available data are still insufficient to establish that IMP can cause colon cancer, which requires further study. Therefore, patients with IMP should be followed up closely to be on the lookout for deterioration and cancer.

**Table 1:** Demographics of patients with idiopathic mesenteric phlebosclerosis in literature review.

Demographics	Value (*n* = 78)	%
Age (years), median (range)	58 (22–84)	
Gender		
Female	37	47.4
Male	41	52.6
Area		
Asia	76	97.4
Europe	1	1.3
Other	1	1.3
Symptoms and signs		
Abdominal pain	59	75.6
Abdominal distension	16	20.5
Constipation	14	17.9
Diarrhea	20	25.6
Nausea or vomiting	21	26.9
Positive stool occult blood	8	10.3
Others	5	6.4
Herb drug history		
Long-term use	58	74.4
No drug history	16	20.5
Not mentioned	4	5.1
Treatment		
Conservative treatment	40	51.3
Surgical intervention	35	44.9
Others	3	3.8
Surgical method		
Total colectomy	14	17.9
Right hemicolectomy	10	12.8
Other hemicolectomy	7	9.0
Ileostomy	4	5.1
Open or laparoscopic surgery		
Open surgery	27	34.6
Laparoscopic surgery	8	10.3

## 4 Conclusion

IMP is a chronic progressive disease that is highly susceptible to misdiagnosis and underdiagnosis in its early stages, when venous sclerosis has not yet developed. Familiarity with the clinical manifestations and imaging features of this disease is important. Further research is needed to explore the specific risk factors and etiology of IMP to reduce its incidence and identify high-risk groups.

## Data availability statement

The original contributions presented in the study are included in the article/supplementary material, further inquiries can be directed to the corresponding authors.

## Ethics statement

Written informed consent was obtained from the individual(s) for the publication of any potentially identifiable images or data included in this article. Written informed consent was obtained from the participant/patient(s) for the publication of this case report.

## Author contributions

SL: Conceptualization, Data curation, Formal analysis, Writing – original draft, Writing – review & editing. YT: Formal analysis, Resources, Software, Writing – original draft. RS: Conceptualization, Resources, Visualization, Writing – review & editing. XZ: Investigation, Methodology, Supervision, Validation, Writing – review & editing. HuaL: Investigation, Writing – review & editing. PY: Formal analysis, Investigation, Writing – review & editing. XC: Project administration, Resources, Writing – review & editing. DW: Data curation, Formal analysis, Funding acquisition, Writing – review & editing. HuiL: Data curation, Investigation, Writing – original draft, Writing – review & editing. JW: Conceptualization, Data curation, Formal analysis, Funding acquisition, Writing – original draft, Writing – review & editing.
